# Development and Usability of ADappt: Web-Based Tool to Support Clinicians, Patients, and Caregivers in the Diagnosis of Mild Cognitive Impairment and Alzheimer Disease

**DOI:** 10.2196/13417

**Published:** 2019-07-08

**Authors:** Ingrid S van Maurik, Leonie NC Visser, Ruth E Pel-Littel, Marieke M van Buchem, Marissa D Zwan, Marleen Kunneman, Wiesje Pelkmans, Femke H Bouwman, Mirella Minkman, Niki Schoonenboom, Philip Scheltens, Ellen MA Smets, Wiesje M van der Flier

**Affiliations:** 1 Alzheimer Center Amsterdam, Department of Neurology Amsterdam Neuroscience Vrije Universiteit Amsterdam, Amsterdam UMC Amsterdam Netherlands; 2 Department of Epidemiology and Biostatistics Vrije Universiteit Amsterdam, Amsterdam UMC Amsterdam Netherlands; 3 Department of Medical Psychology Amsterdam Public Health Research Insitute University of Amsterdam, Amsterdam UMC Amsterdam Netherlands; 4 Vilans Center of Expertise for Long Term Care Utrecht Netherlands; 5 Medical Decision Making Department of Biomedical Data Sciences Leiden University Medical Center Leiden Netherlands; 6 Tilburg University TIAS School for Business and Society Tilburg Netherlands; 7 Department of Neurology Spaarne Gasthuis Haarlem Netherlands

**Keywords:** Alzheimer’s disease, biomarkers, decision aids, mild cognitive impairment, precision medicine, risk, shared decision making

## Abstract

**Background:**

As a result of advances in diagnostic testing in the field of Alzheimer disease (AD), patients are diagnosed in earlier stages of the disease, for example, in the stage of mild cognitive impairment (MCI). This poses novel challenges for a clinician during the diagnostic workup with regard to diagnostic testing itself, namely, which tests are to be performed, but also on how to engage patients in this decision and how to communicate test results. As a result, tools to support decision making and improve risk communication could be valuable for clinicians and patients.

**Objective:**

The aim of this study was to present the design, development, and testing of a Web-based tool for clinicians in a memory clinic setting and to ascertain whether this tool can (1) facilitate the interpretation of biomarker results in individual patients with MCI regarding their risk of progression to dementia, (2) support clinicians in communicating biomarker test results and risks to MCI patients and their caregivers, and (3) support clinicians in a process of shared decision making regarding the diagnostic workup of AD.

**Methods:**

A multiphase mixed-methods approach was used. Phase 1 consisted of a qualitative needs assessment among professionals, patients, and caregivers; phase 2, consisted of an iterative process of development and the design of the tool (ADappt); and phase 3 consisted of a quantitative and qualitative assessment of usability and acceptability of ADappt. Across these phases, co-creation was realized via a user-centered qualitative approach with clinicians, patients, and caregivers.

**Results:**

In phase 1, clinicians indicated the need for risk calculation tools and visual aids to communicate test results to patients. Patients and caregivers expressed their needs for more specific information on their risk for developing AD and related consequences. In phase 2, we developed the content and graphical design of ADappt encompassing 3 modules: a risk calculation tool, a risk communication tool including a summary sheet for patients and caregivers, and a conversation starter to support shared decision making regarding the diagnostic workup. In phase 3, ADappt was considered to be clear and user-friendly.

**Conclusions:**

Clinicians in a memory clinic setting can use ADappt, a Web-based tool, developed using multiphase design and co-creation, for support that includes an individually tailored interpretation of biomarker test results, communication of test results and risks to patients and their caregivers, and shared decision making on diagnostic testing.

## Introduction

Dementia is a syndrome diagnosis that is used to describe decline in cognitive functioning that is severe enough to result in a loss of independence in performing everyday activities [1]. A growing proportion of individuals presenting at memory clinics do not (yet) fulfill the criteria for dementia. These individuals are labeled as having mild cognitive impairment (MCI) if cognitive impairment can be objectified [2]. In the course of 3 years, roughly half of the MCI patients develop dementia, whereas the other half remain stable or improve [3]. Therefore, the label of MCI entails a prognosis, rather than a diagnosis.

The prognosis of MCI patients depends on the etiology of the symptoms. The most common underlying cause is Alzheimer disease (AD), a neurodegenerative disease that develops gradually, with dementia as the final stage [4]. AD biomarkers, assessed through, for example, magnetic resonance imaging (MRI) or cerebrospinal fluid (CSF), reflect AD related pathological processes and can therefore provide information on the underlying cause of cognitive impairment [5]. In early stages of the disease, AD biomarkers are particularly valuable, as this information allows a more precise estimate of the patient’s risk of developing dementia [6]. However, interpreting and communicating biomarker results is complex, as the understanding of probabilistic information is known to be difficult for clinicians and patients and caregivers [7]. Moreover, test results may be unclear or conflicting and thus may not always offer the certainty that clinicians, patients, and caregivers are looking for [8].

As a result, the clinician has to face a growing number of challenges during the diagnostic workup, for example, which and how many diagnostic (biomarker) tests should be performed, how much and what type of information does the patient actually want, what can we expect from a specific diagnostic test, does a patient prefer to be provided with information on the likely course of their symptoms, and how can this information best be conveyed. Given that patients may weigh potential benefits and harms of AD biomarker testing differently, considering the patients’ preferences and needs are essential when making decisions for or against biomarker testing and the disclosure of results. In this situation, where an increasing number of reasonable options emerge to address the patients’ situation, clinicians and patients should engage in a process of shared decision making to ensure that the decisions made best fit the individual [9]. However, in a memory clinic setting, involving patients in decision making is often limited to providing information only [9,10], and explicit risk communication about the development of dementia is barely observed in individuals with MCI. This demonstrates that there is room to support clinician-patient communication [9].

In the context of the Alzheimer Biomarkers in Daily Practice (ABIDE) project, we aimed to develop a Web-based tool for clinicians in a memory clinic setting that (1) provides personalized risk estimates of progression to dementia to aid the clinician in interpreting test results, (2) supports clinicians in communicating biomarker test results and risks to MCI patients and their caregivers, and (3) supports clinicians to engage patients in decision making regarding the diagnostic workup of AD. Following a user-centered design, this paper describes the process of development, usability, and acceptability of the Web-based diagnostic support tool named ADappt.

## Methods

### Study Design

This study was conducted as part of the ABIDE project, which has been funded in the context of the Dutch Deltaplan Dementia [11]. ABIDE has been designed to improve and facilitate the use and interpretation of AD biomarkers in clinical practice, taking into account patients’ preferences toward diagnostic testing and communication of test results. Here, we describe the development and testing of the usability and acceptability of ADappt, a Web-based tool to support clinicians in the diagnostic process of AD in patients with MCI. The tool consists of 3 modules: (1) risk calculation: this module provides personalized risk estimates of progression to dementia to aid the clinician in interpreting test results (MRI and CSF); (2) risk communication: this module provides a summary of the biomarker results, including a graphic representation of the risk to facilitate communication of test results; (3) shared decision making: this module entails a conversation starter to support clinicians to engage patients in shared decision making regarding diagnostic testing.

**Figure 1:**
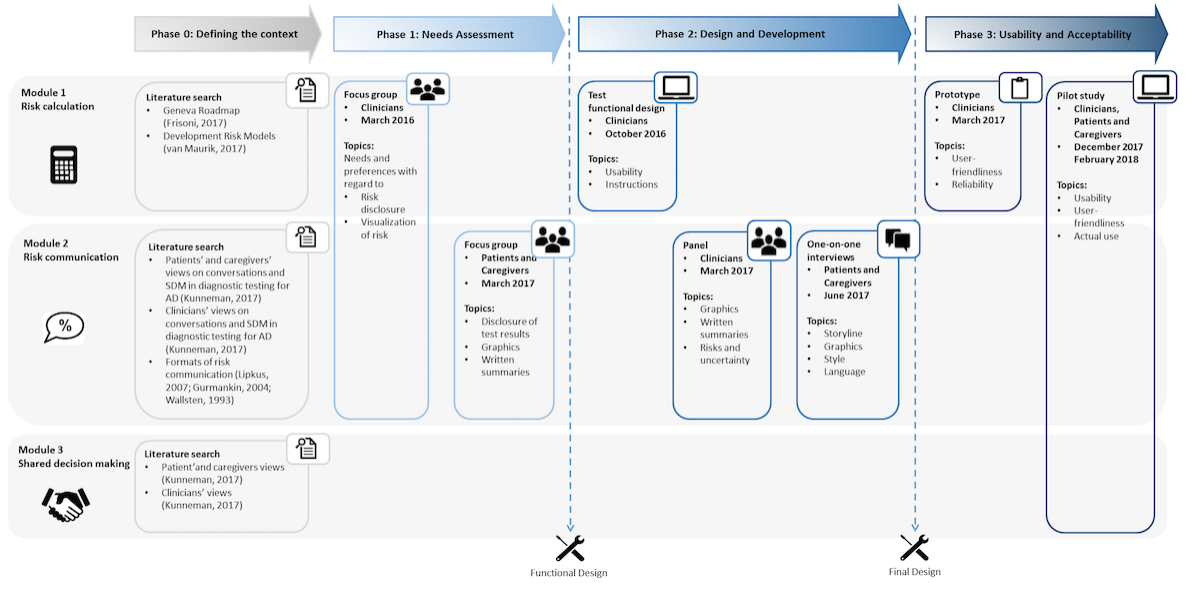
Study overview. AD: Alzheimer disease; SDM: shared decision making.

The overall study adopted a user-centered mixed-method approach with emphasis on co-creation and was conducted in the 3 phases described in Figure 1. The board of the medical ethics review committee of the Vrije Universiteit (VU) medical center reviewed and approved this study. For each module, input and feedback of end users (professionals, patients, and caregivers) was obtained. Modules were developed in independent yet overlapping trajectories such that module 1 was developed first, closely followed by module 2. Module 3 was developed last, based on a set of earlier studies conducted in the context of the ABIDE project [9-10,12].

Thematic content analysis was used to analyze the qualitative data collected during focus groups, panels, and one-on-one interviews [13]. In total, 2 coders (IvM and LV), with a background in psychology, used an inductive approach to independently formulate lists of themes that appeared from summarized data. The themes were discussed to reach consensus.

### Module 1: Risk Calculation

#### Phase 0: Defining the Context

In 2016, the Geneva Task Force on the roadmap of Alzheimer biomarkers published a strategic roadmap to foster the clinical validation of AD biomarkers [14]. This roadmap adopted a 5-phase approach from cancer research and dedicated 1 phase to the diagnostic and prognostic performance of biomarkers in MCI patients. Both for MRI and CSF biomarkers, evidence still appeared incomplete [15,16]. In particular, limited evidence was available on the combination of biomarkers and how they perform to predict prognosis in individual MCI patients. In a previous study, we therefore constructed individualized prediction models that provide personalized risk estimates for MCI patients, on the basis of MRI, CSF, or the combination of these 2 biomarkers based on data from the Amsterdam Dementia Cohort [6,17,18]. Probabilities (with CIs) of progression to AD dementia for 1 and 3 years of follow-up can be calculated using these models (for a detailed description see van Maurik et al [6]). These models serve as input for the risk calculation module of ADappt.

#### Phase 1: Needs Assessment

##### Focus Group Clinicians

We organized a 2-hour focus group (n=8, 7 neurologists and 1 geriatrician) led by experienced focus group leaders (RP and MK). During this focus group, we discussed the interpretation of biomarker test results and communication of results in terms of risks to patients based on 2 clinical cases that were shortly introduced. Used examples of risk visualizations were based on a commonly used format in cardiovascular risk management [19] and breast cancer therapy risks (Adjuvant! Online) [20].

#### Phase 2: Design and Development

##### Test Functional Design

A prototype of the risk calculation module (module 1) was developed from the results of phase 1. Functionality of the initial design was tested by end users in 2 rounds. In both rounds, end users were asked, via a questionnaire, if they considered the module to be logical, clear, and useful. In the first round, a neurologist, a geriatrician, and 2 researchers provided input on a preliminary design and layout (risk calculation functionality was not yet available). From their input the prototype was further developed. Next, 9 professionals (5 neurologists, 2 geriatricians, and 2 health care professionals) provided input on the prototype with a fully functioning risk calculation.

#### Phase 3: Usability and Acceptability

##### Questionnaire Feedback Prototype

During the annual Dutch Dementia Conference (Dementia Update) in 2017, the risk calculation module was presented and interested attendees were given the opportunity to try out the prototype. A total of 24 attendees (4 geriatricians, 10 neurologists, 5 with another profession [internist or nursing home physician specialist], and 5 did not indicate their profession) provided written feedback by filling out a brief questionnaire.

### Module 2: Risk Communication

#### Phase 0: Defining the Context

In a previous survey study [10], clinicians indicated that they find it difficult to convey MCI as a diagnosis because of the uncertainty of the diagnosis of MCI and the lack of treatment. Nonetheless, the majority of clinicians indicated to always disclose the risk of developing dementia [10]. This however does not sufficiently fulfill the information need of patients and caregivers, as patients expressed a wish for more information on the prognosis of the disease [12].

In risk communication, numeric formats are generally preferred, relative to other formats, to increase patients’ understanding [7,21,22]. For example, verbal communication of risk (unlikely, possible, and rare) is vulnerable to a high degree of variability in interpretation and therefore not considered a best practice [7]. Among numeric formats, natural frequencies (20 out of 100 people similar to you) are favored over other formats (probabilities, odds, or classical probabilities), as a reference class is included that reduces misinterpretation [7,23]. A numeric format should ideally be complemented with a visual representation of risk and include a specific time frame in which an event may occur [24].

#### Phase 1: Needs Assessment

##### Focus Group Clinicians

Input from clinicians with regard to risk communication was obtained in the same focus group as described in phase 1 for module 1 (see the section above for details).

##### Focus Group Patients and Caregivers

A total of 4 1-hour focus groups were organized for MCI patients and were led by an experienced focus group leader (RP). A total of n=13 patients participated (3 to 4 participants per session, mean age 66 years (SD 8); 31% (4/13) female) and 63% (8/13) were accompanied by a caregiver (all partners, n=4 (50%) female). The topics that were discussed included how patients and caregivers looked back on receiving the diagnosis and test results and whether they had any recommendations for the disclosure of test results. In a second part, participants brainstormed hands-on in a paper, pencil, and scissors session on useful (graphical) summaries of test results.

#### Phase 2: Design and Development

##### Panel Clinicians

During the annual Dutch Dementia Symposium (Dementia Update) in 2017, clinicians were invited to attend a short panel discussion on their preferences about tools to support communication of test results, in terms of graphics, written summaries, and explanations of risks and uncertainty. A total of 5 clinicians attended this panel discussion and provided feedback.

##### Individual Interviews With Patients and Caregivers

To evaluate the first prototypes of the risk communication module, we conducted 5 interviews, each with 1 MCI patient (mean age 65 years [SD 9], n=2 (40%) female) and his or her caregiver (all partners, n=3 (60%) female). Interviews took 15 to 30 min and were conducted by the software developer to ensure that the feedback of patients was optimally used to improve this module (co-creation). Patients were presented with an example of the module, in which the diagnostic test results of a fictive case of a man or woman of 60 years were displayed, and patients were asked for their input on the storyline, graphics, style, and the possibility to print these results.

### Module 3: Shared Decision-Making Module

#### Phase 0/1: Defining the Context/Needs Assessment

In shared decision making, clinicians and patients work together to decide which care plan best fits with individual preferences and needs when there is more than 1 reasonable option [25,26]. In the diagnostic routine for AD, there is typically more than 1 reasonable option to choose, making this a situation where shared decision making is the preferred approach. However, in a previous study, we found that shared decision making in the memory clinic is often limited to information giving [10], and shared decision making involves the following 4 steps: (1) create choice awareness, (2) provide information, (3) explore preferences, and (4) decide together [27,28].

#### Phase 2: Design and Development

For each step in shared decision making, we constructed example phrases in co-creation with communication experts (LV and ES). These phrases might function as a conversation starter for clinicians to engage patients in shared decision making. To inform a patient on the possible pros and cons, we also developed a list with the example language on (dis)advantages for the following commonly used diagnostic tests: neuropsychological investigation, imaging (MRI or computer tomography [CT]), lumbar puncture, amyloid Positron Emission Tomography (PET), consultation with other specialist (neurologist, geriatrician, and psychiatrist), clinical geneticist, and awaiting policy. This list was based on the literature and expert opinions (FB and WF).

### All Modules: Pilot Study in 4 Local Memory Clinics

To test the ADappt tool comprising all 3 modules, we organized a multicenter usability pilot study. Both clinicians and patients were asked for feedback in 4 local memory clinics (Reinier de Graaf Ziekenhuis, Elisabeth Tweesteden Ziekenhuis, Jeroen Bosch Ziekenhuis, and Spaarne Gasthuis) and 1 academic memory clinic (Amsterdam University Medical Center (UMC)). Participating clinicians (4 neurologists, 1 geriatrician, and 2 not specified) were asked to use the tool for a minimum of 2 weeks and a maximum of 7 weeks. One of the authors (MvB) was present in the memory clinics to assist clinicians in the first week of the pilot. Clinicians were asked to complete the System Usability Scale (SUS) after using the tool [29]. The maximum score on the SUS is 5, and a higher score indicates better usability. Technical details of the tool are summarized in Textbox 1.

**Technical development of the tool.**

The tool was constructed as a responsive Web app, meaning that the tool is available on every device (desktop, tablet, and mobile phone) and is developed in the React framework (hypertext markup language [HTML]/cascading style sheets [CSS]/JavaScript). Hosting is managed by Acato and delivered by Oxillion. Data entered into the tool are not saved in a database to minimize privacy related issues. The tool is compatible with Internet Explorer (10 and 11), Edge (13 and 14), Firefox (50 and 51), Chrome (56 and 57), iPhone operating system (iOS) Safari (9.3 and 10), and Android browser (4.4 and 4.4.4). The tool is located at [30,31]. The risk calculator is not yet available for medical use, but a login for academic use can be provided by the authors or via the contact form on the website [30]. The tool is Conformité Européenne certified (self-certification) in the lowest risk class (B).

## Results

### Module 1: Risk Calculation Module

**Figure 2:**
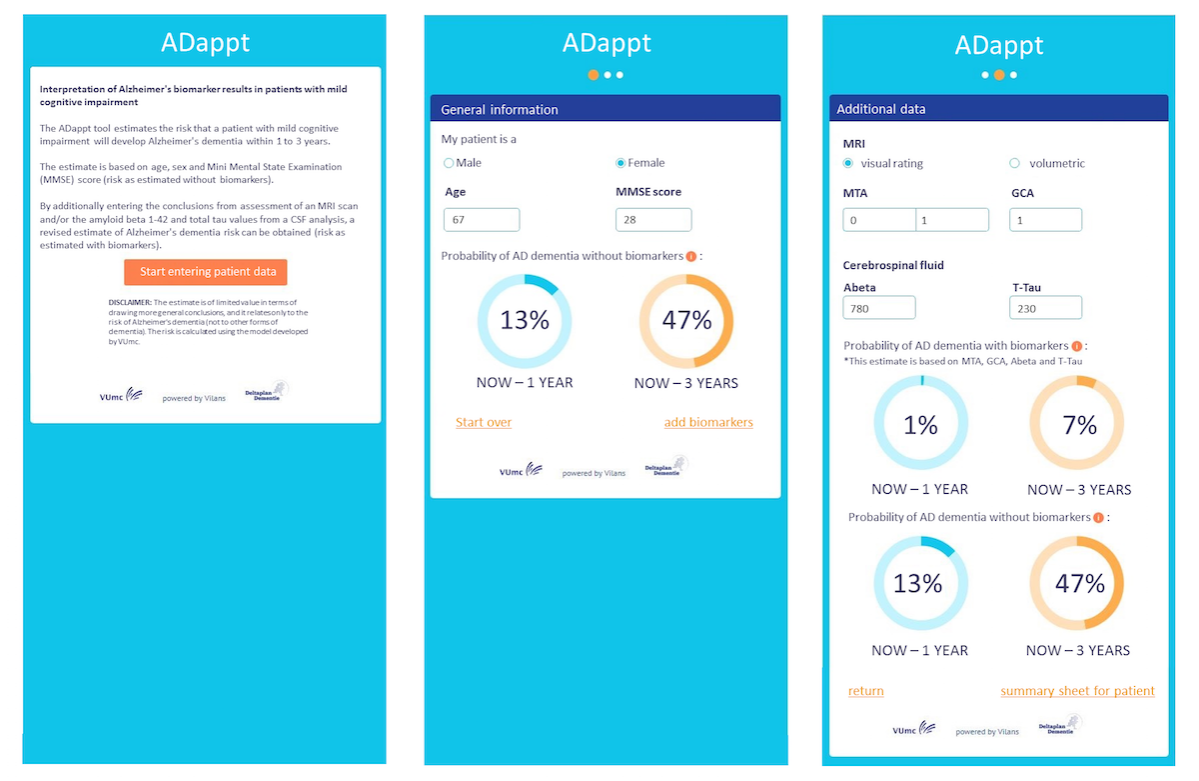
Final design of the risk calculation module (module 1).Left: start page with disclaimer. Middle: risk calculation module based on demographic information only (probability of AD dementia without biomarkers). As an example, we entered data of a fictional female MCI patient, age 67 years and mini mental state examination (MMSE) score of 28, resulting in a 1 year probability of progression of 13% and 3 year probability of 47%. Right: adding biomarkers for the same patient; Hippocampal atrophy (visually rated with Medial temporal lobe (MTA) atrophy scale), global cortical atrophy (GCA), Abeta, and and total tau. In this case, the normal biomarkers (the tool calculates with continuous data) resulted in a strong decrease of progression probabilities. To appreciate the change in probabilities, the tool repeats the initial risk based on demographic data at the lower part of the module.

Here, we describe the results of the co-creation steps leading to the final layout of the risk calculation module shown in Figure 2.

#### Phase 1: Needs Assessment

##### Focus Group Clinicians

On reviewing the discussion on the preferences with regard to risk calculation, 3 main findings emerged (the findings and adaptations are presented in Table 1). First, clinicians preferred the reporting of percentages over a risk table or bar chart, as in the latter, the information was perceived not to be clear at one glance. Second, regarding reliability and validity; clinicians whished information about CIs and information on how the models were constructed and how they perform. Therefore, we included a link to the publication, explaining how the models were constructed. Finally, clinicians emphasized user-friendliness of the module, for example, every professional should be able to use the module.

**Table 1:** Overview of themes for each focus group.

Group		Aim	Themes	Changes made based on the results
**Focus group**	
	Clinicians	To explore the need for risk calculation and communication and preferences with regard to visualization	Module 1: Risk calculation. Reporting percentages is considered pleasant.; Reliability and validity; CIs should be added and it should be possible for clinicians to look up how the risk models were constructed and how they perform; The module should be user-friendly such that every professional is able to use it.; Module 2: A graphical representation of risk alone could be difficult for patients to understand and therefore would not be sufficient; Clinicians often show brain images to support the communication of test results; Clinicians provide patients with written information.	We included a link to the original paper that describes how the risk models were constructed; We accompanied the graphical presentation of risk with a textual explanation; The module allows summary sheet for patients to be printed
	Patients and caregivers	To explore communication needs and to explore what kind of information should be communicated when disclosing (risk related) test results and how it should be visualized	Module 2: Risk Communication. Patients would like to know their exact progression risk. The explanation of risk should be simple. To compare their results with what is normal for their age. How should I discuss the results with family or friends?; Visualization: Patients like to see their brain images (MRI^a^ or CT^b^) at home. Patients like to know which results have led to the presented risk and why. The preference for visualization of risk differs per person. How should I deal with the diagnosis? Are there things that I can do myself (lifestyle changes, tips and tricks)? Which steps do we need to take (case manager etc)?	We included an *a priori* risk as reference group for comparison purposes. The result page includes information on each diagnostic test and whether this result was abnormal. A summary is given at the end with the opportunity to include personal notes. We included 2 ways to visualize risk: a bar chart and an icon array. Users can switch between these 2
**Panel**	
	Clinicians	To explore the preferences with regard to visualization of test results for patients	Clinicians like to communicate as *dichotomous’* as possible. However, biomarker results always have a gray area. This could be visualized by a *traffic light*; Clinicians would like to visualize risk in a line chart with time on the horizontal axes; The brain images (MRI or CT) could be visualized with a figure of the brain: normal versus atrophy; Clinicians emphasize that the module should be an aid, and that there should remain room for personal variation	We added a traffic light visualization for all diagnostic tests; An open text field provides the ability to personalize the result page based on the needs of clinicians and patients
**Individual**	
	Interviews	To explore whether the storyline, visualization, and style of the printout page are clear	The printout contains too much and too complex information; The presentation of the *a priori* risk is confusing and sometimes daunting; Patients prefer to see their test results first followed by the risk; The graphical presentation of risk in 2 ways works well; The font is too small; The print of the results page is considered as valuable.	A total of 2 communication experts reviewed the text on understandable language; We removed the *a priori* risk from this module; We changed the flow of the printout: test results are presented first followed by risk; Colors and font were optimized

^a^MRI: magnetic resonance imaging.

^b^CT: computer tomography.

#### Phase 2: Design and Development

##### Test Functional Design

End users agreed on the proposed order and name of the tool. In general, the information in the module was considered to be clear. Suggestions for improvement included (1) a clear disclaimer that the module calculates risk of AD dementia only, (2) to use a different term instead of a priori and a posteriori risk, (3) adding value ranges, and (4) the module should also work when only CSF or MRI values were entered.

#### Phase 3: Usability and Acceptability

##### Questionnaire Feedback Prototype

The responses of the n=24 clinicians are summarized in Table 2. The applicability and user-friendliness of the module was rated highest. More than half of the respondents indicated that they would use the final version of the module in the future. The perceived reliability of the module was rated relatively low.

**Table 2:** Overview of first round of usability rating.

Questions	Rating
Is it clear where ADappt could be used for (rate 1-10)?	8.3
How user-friendly would you rate this tool (rate 1-10)?	8.3
How reliable would you rate ADappt (rate 1-10)?	6.4
Would you use the final version of ADappt in your daily clinical routine (% yes)?	58%

### Module 2: Risk Communication Module

**Figure 3:**
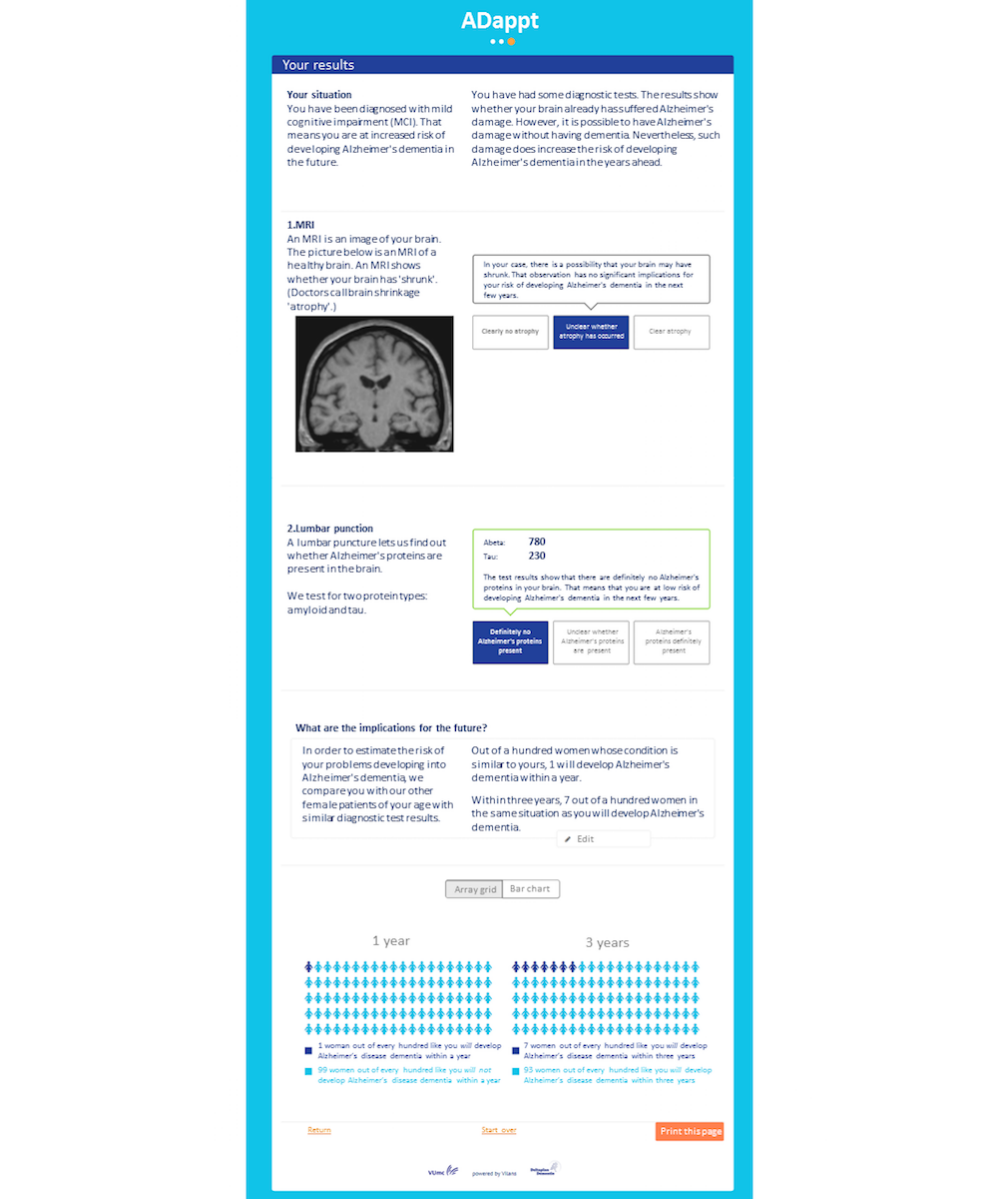
Final design of the risk communication module (module 2). Here we use the same example as in Figure 2. First, a general description of MCI is given, followed by the personalized results of this person. For each test, we give a summary of what kind of information is retrieved from the test, followed by the interpretation of the test result and how it effects the chance of AD dementia. Finally, we summarize the findings and give a verbal explanation (1 out of 100) of risk and visualization of this risk, either with an array grid or bar chart (not shown). Of note, the MRI is a stock image, not personalized.

To facilitate explanation of the test results, we developed in co-creation (steps described below) a risk communication sheet. The final layout is shown in Figure 3. The final design of the summary sheet (module 2). Here we use the same example as in Figure 2. First, a general description of MCI is given, followed by the personalized results of this person. For each test, we give a summary of what kind of information is retrieved from the test, followed by the interpretation of the test result and how it effects the chance of AD dementia. Finally, we summarize the findings and give a verbal explanation (1 out of 100) of risk and visualization of this risk, either with an array grid or bar chart (not shown). Of note, the MRI is a stock image, not personalized.

#### Phase 1: Needs Assessment

##### Focus Group Clinicians

With regard to risk communication, clinicians mentioned that they often show brain images to patients to support communication of test results and that they provide patients with written information (findings and adaptations are presented in Table 1). In addition, clinicians confirmed that a graphical representation of risk only is not sufficient to explain a risk to patients and caregivers. Therefore, we accompanied the visual presentation of risk with a textual explanation.

##### Focus Group Patients and Caregivers

An important theme during the focus group with patients and caregivers was that patients would like to know their exact progression risk. Other themes with regard to risk were that explanations should be simple and that they would like to compare their results with a normal reference category. In addition, patients indicated to struggle with how they should communicate their diagnosis and test results with family and friends (Table 1). In terms of visualization, patients preferred to see their own brain images. However, in view of ever-more complicated privacy regulations, we decided not to include this in the summary sheet. Patients would also like to know how the test results contributed to the presented risk. Individual differences emerged with regard to the preference for the visualization of risk (bar chart or icon arrays). Therefore, we decided to incorporate both options in the module, to be used depending on patients’ preference. Finally, patients and caregivers would like to be provided with information on how to handle the diagnosis, what they can do in terms of lifestyle changes and which steps they should take next (for example a case manager). As this is likely to be different for each patient, we included an open field at the end of the summary sheet that can be used by the clinician to include such information.

#### Phase 2: Design and Development

##### Panel Clinicians

One of the themes emerging from the panel discussion with clinicians is that they prefer to communicate test results in a dichotomous way (normal or abnormal, Table 1). However, biomarker results are originally continuous in nature, and in terms of interpretation, there is always a gray area. A possible way to visualize this is by using a traffic light. Therefore, we included a traffic light for all biomarkers on the summary sheet, with red indicating abnormal, green indicating normal, and gray indicating borderline results. As an alternative to including the MRI or CT images of patients, clinicians suggested to implement an example MRI image on the written summary for patients. For risk visualization, clinicians suggested to use a line chart with time on the horizontal axis. However, as the risk calculation module only provides valid predictions up to 3 years, a line graph might incorrectly imply that this line could be extrapolated to longer follow-up periods. For this reason, we decided not to make use of this suggestion. Instead, we included 2 options for risk visualization: a bar chart and an icon array. Finally, clinicians emphasized that the communication module should be an aid, allowing for tailoring of the communication to individual patients, rather than a strict protocol. This whish is met by providing an open text field in the summary sheet, where the clinician can add information.

##### Individual Interviews

During the individual interviews with patients and caregivers, the storyline of the printout was considered as too long and too complex. Therefore, 2 communication experts reviewed, optimized, and abbreviated the text (LV and ES). Moreover, we removed the a priori risk (ie, risk without biomarkers, see example in Figure 2) from the summary sheet for patients as this was experienced as confusing and sometimes daunting. Patients and caregivers only valued the probability based on the diagnostic tests. Patients would like to see their individual test results first, followed by their overall risk as a result of these individual test results. The graphical presentation of risk as either a bar chart or icon array worked well and the printout page was considered valuable. Themes and adaptations are summarized in Table 1.

### Module 3: Shared Decision-Making Module

#### Phase 2: Design and Development

Table 3 presents the conversation starter with example phrases for a clinician to engage patients and caregivers in each of the 4 steps of shared decision making (create choice awareness, provide information, explore preferences, and decide together [27,28]). In Table 4, we show example language that can be used to inform a patient on pros and cons with regard to imaging. Phrases for other diagnostic tests can be found on our website [32].

**Table 3:** Example phrases to start shared decision making.

Step	Aim	Example
Creating choice awareness	To make the patient aware that there is more than 1 way forward, that a decision needs to be made on whether and which diagnostic test to use, and that the best way forward depends on what matters most to the patient	“The aim is that we decide (together) which diagnostic test we will perform.”
Provide information	To assess patients’ preferences	“There is much to tell about the different diagnostic tests. Some patients would like to hear as much as possible, while other patients do not want to know too much. What do you prefer?”
	To inform on the different diagnostic test possibilities	“What would you like to know about the possibilities for diagnostic testing?”; or “There are 3 possibilities, namely…”
	To inform on the pros and cons in general or for a specific test	“Would you like to know more about the possible pros and cons?”
	To inform which results can be expected	“I will tell you something about the kind of results that you can expect when we perform a specific test”
Explore preferences	To explore preferences and considerations from the patient	“I have told you about the possibilities, what are your considerations?”
	To estimate the decision preferences of the patient. Does the patient want to be involved in the decision or does the patient want the clinician to decide	“Some patients would like to decide with the clinician which test to perform, while others would like the clinician to decide. What do you prefer?”
Decide	To make a shared decision; a balanced decision that both parties support, and communicate this	“We decide to do [X], because you indicated that [preferences patient].”
	To formulate an advice, in which the preferences of the patient are taken into account, and communicate this	“I propose [X], based on [preferences patient, guidelines, experience, preferences clinician]. Do you agree?”

**Table 4:** Example phrases for magnetic resonance imaging (MRI) and computed tomography (CT).

Reason why MRI or CT scan is suggested	Pros	Cons
“A scan of the brain is a common part of dementia diagnostics. With MRI^a^ or CT^b^ scan we visualize the brain. We can exclude treatable diseases. Moreover, we can view characteristics of dementia, such as shrinkage of the brain or wearing of the blood vessels.”	“Detecting treatable diseases, such as a brain tumor, brain infarct, or hemorrhage.” “Detecting characteristics of dementia.” “An MRI^a^ scan shows more details than a CT^b^ scan.” “A CT^b^ scan is less cumbersome as the scan takes less time (10-15 min) and the CT^b^ scanner is more spacious. Also, a pacemaker is not a problem.”	“A patient may not move for 30 min in the MRI^a^ scanner.” “For some patients an MRI^a^ scan may be an anxious experience (claustrophobia).” “It is not always possible to perform an MRI^a^, for example, if a patient has a pacemaker.” “A CT^b^ scan shows les details than an MRI^a^ scan.”

^a^MRI: magnetic resonance imaging.

^b^CT: computer tomography.

### Pilot Study in 4 Local Memory Clinics

**Figure 4:**
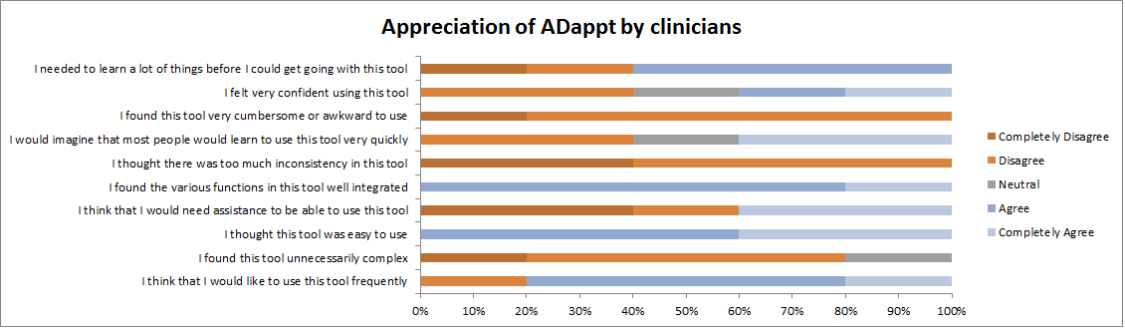
Overview of pilot results: responses of clinicians to each question of the system usability scale.

In total, 7 clinicians from 5 memory clinics commented on the ADappt tool and 5 of them completed the survey. The usability of the tool was rated with a score of SUS=4.4 (SD 0.5, max score=5; see Figure 4). Moreover, clinicians rated the tool as well integrated, easy, and consistent. A total of 3 clinicians used the tool in their daily practice, of which 1 used it on a regular basis during the pilot. Figure 1 summarizes the response frequencies of each question in the survey:

[…] it was very useful, because this patient had somewhat conflicting biomarkers, which did not point in one direction, so when using the app, it resulted in a much lower risk than that these people expected, and actually they went home very satisfied.

[…] I did use the website, but mainly the result page to provide the patient with more insight in what it could mean when it results in MCI and what the risk on dementia eventually is and that you can give them a printout to take home, that is very positively received also by the patient.

Clinicians experienced some technical problems while using the tool. For example, the site was blocked by the firewall in 1 of the hospitals, leading to the need to use it on a personal device. Some clinicians commented that they already feel time pressure during a consult and do not find the time to actually open the tool in the consult. A total of 4 patients (n=3 (75%) female, mean age 72 years [SD 2]) completed a short questionnaire after the consult in which the tool was used. The result page was valued as clear and useful by all. The result pages helped them to better understand the diagnosis and helped them explain the results to relatives and/or friends. These patients recommended the result page to other patients.

## Discussion

This study reports the development and initial usability testing of ADappt—a Web-based tool to facilitate risk calculation and support clinician-patient risk communication and shared decision making in a memory clinic setting. We worked closely with end users, clinicians, patients, and caregivers, to identify initial requirements for the tool and make necessary changes to enhance the functionalities, usability, and information provided by the tool. Needs assessment (phase 1) uncovered the need of patients and caregivers for written summaries and information on prognosis. Clinicians expressed their need for visual aids to communicate results and prognosis. In the development phase (phase 2), we developed a risk calculation tool for clinicians and a risk communication tool (summary sheet) for patients and caregivers. In addition, a conversation starter was developed to support clinicians and patients in decision making around diagnostic testing.

Already in an early stage of usability testing (phase 3), the tool was considered clear and user-friendly, and a majority of memory clinic professionals indicated they would use such a tool in the future. However, clinicians appeared to have concerns about the reliability of the risk prediction tool. At the moment of testing, the algorithms were not yet published, and therefore a reference to a detailed description of the models was not included in the tool. To date, the module has been updated with the published algorithms [6]. For the tool to be used in real clinical practice, generalizability of prediction models in the risk calculation module is essential. Therefore, we are generating updated risk models based on data from a range of multi and monocenter cohorts and in alignment with the recently published National Institute on Aging and Alzheimer’s Association (NIA-AA) criteria [5]. These updated models will be applicable to CSF biomarkers and volumetric MRI markers measured with different assays and platforms. In addition, we will add models based on amyloid PET. Another short-term update will be the translation of the tool into English.

Our small pilot study demonstrated that the tool, especially the printout page (risk communication module), was considered valuable by patients and caregivers. Although the usability of the tool was rated to be high by clinicians, only few clinicians actually used the tool in clinical practice. This was mainly due to practical reasons; clinicians experienced technical hurdles for the use of such a tool in clinical practice (eg, computers in consulting room firewalled). Despite these hurdles and that relying on computer algorithms may be counterintuitive for clinicians, experience from different disease areas shows that computer algorithms are welcome in clinical practice and are seen as the future of medicine. For example, in oncology clinicians use Adjuvant Online to calculate the risk of recurrence with and without adjuvant therapy for breast cancer patients [20]. Recently U-Prevent was introduced for clinicians to calculate cardiovascular risk and therapy benefit [33,34]. The major challenge for ADappt is its large-scale implementation. In an ideal situation, ADappt would be embedded in electronic patient file systems. Alternatively, ADappt can be used as a simple add-on help for both the clinician and patient to be quickly consulted on their mobile phone. In the short term, an e-learning could be useful to show how the clinicians can integrate the tool in their routine. Moreover, the amount of text in the tool was still experienced as a barrier by clinicians and could be further improved by using graphics instead of plain text.

The 3 modules of ADappt could aid clinicians and patients in a number of ways. In the diagnostic process for AD, it is currently not common practice to actively engage patients in diagnostic decision making [10,25]. ADappt provides a conversation starter and an overview of pros and cons per diagnostic test and could therefore help clinicians to engage patients and caregivers in diagnostic decision making [9]. In an observational study of ABIDE, we currently examine communication and decision-making processes during pre- and postdiagnostic clinician-patient encounters. This study will provide input to further improve the shared decision-making module, as soon as the results of this study become available [11].

Second, ADappt supports clinicians in the communication of test results to patients and contributes to personalized diagnostic care and harmonization of clinical practice. After diagnostic testing, the interpretation and communication of test results, especially when patients are not yet demented (ie, MCI) is not straightforward. The ADappt tool clearly shows whether a patient is unlikely to develop AD dementia in the following years and therefore could be reassured, or whether a patient is indeed likely to convert to AD dementia and should therefore be followed up, arranged care, or referred for participation in clinical trials. The importance of an appropriate diagnosis and prognosis of MCI is acknowledged in current guidelines. These guidelines state that an appropriate diagnosis of MCI is important for patients and caregivers to understand the cause of their complaints and to arrange care planning based on communicated prognostic probabilities [35]. Moreover, clear communication of the test results and the corresponding prognosis has been given high priority by patients and caregivers. Tools similar to ADappt can help in this regard. One of the strengths of this study is that we designed and co-created ADappt with the end users, that is, clinicians, patients, and caregivers in memory clinic settings, to optimize future use of the tool. Most suggestions and whishes were met in the design and development of the tool. Only 2 suggestions did not find their way into the tool immediately. First, a recurring theme was the use of CT or MRI images to support the communication of diagnosis and test results. Both clinicians and patients indicated that they would like to see a patients’ CT or MRI image to be included on the summary sheet. However, this would require the summary sheet to make a link to, for example, the electronic patient file that is challenging, both from a technical and privacy perspective. As an alternative, we suggested that the clinician shows the patient their own MRI on screen in the consulting room, and we included an example MRI on the summary sheet. Second, in early phases of the development of the summary sheet, patients indicated that they would like to see their results in comparison with a reference class (ie, what is normal for my age). In the first design of the summary sheet, this reference class was presented as separate probabilities and patients could compare this with their personal risk. However, this led to confusion and patients suggested that this should be removed from the summary sheet. Another strength of the tool is its compatibility with the most recent versions of the most commonly used Web browsers and could be used on all commonly used devices (computer and mobile phone). Moreover, the tool is designed as a flexible platform with the idea that extra modules can easily be added in the future. This would also enable adding modules on lifestyle advice [36], extension to other biomarkers or settings, such as the general practitioner, or to advanced care planning.

Among the limitations is that other factors, such as cardiovascular disease on MRI, cardiovascular risk factors, and amyloid PET biomarkers are currently not accounted for in the risk prediction. Clinicians do consider these factors to be important for prognosis in risk prediction and should therefore be accounted for in the models. However, the algorithms incorporated in the tool had a different starting point—namely, how to extract maximum information from MRI and CSF biomarker values for an individual patient, given that the clinician decided to order these tests. For a user-centered development process, the number of participants in this project was adequate. However, to achieve more generalizable results with regard to implementation or cost-effectiveness, future validation should include larger samples and a different study design.

In conclusion, this study presents the first tool to support clinicians and patients in memory clinic settings with decisions on diagnostic testing, individual tailored interpretation of diagnostic test results, and communication of test results. At the moment, the tool is available for academic use only. The tool is developed in a multiphase design, where co-creation with end users was an important feature. Owing to its flexibility, it is possible to add extra modules, guidelines, or new prediction models in the future. Moreover, as the tool currently focuses on clinicians, we envision that in the future a similar platform would be valuable for patients and caregivers to facilitate them to engage in shared decision making and to aid them in managing their own health care trajectory.

## Abbreviations

ABIDE: Alzheimer biomarkers in daily practice
AD: Alzheimer disease
CSF: cerebrospinal fluid
CSS: cascading style sheets
CT: computer tomography
MCI: mild cognitive impairment
MRI: magnetic resonance imaging
PET: positron emission tomography
SUS: system usability scale
